# Supplementary material for: Modular automated bottom-up proteomic sample preparation for high-throughput applications
Source: PLoS One. 2022 Feb 25;17(2):e0264467. doi: 10.1371/journal.pone.0264467 (PMC8880914; doi:10.1371/journal.pone.0264467)
Supplement: S1 File — Also available on protocols.io. (PDF) [file pone.0264467.s001.pdf]

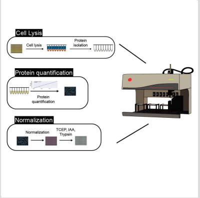

2

Jan 12, 2022

Modular automated bottom-up proteomic sample preparation for high-throughput applications V.2

Yan Chen[1], Nurgul Kaplan Lease[1], Jennifer Gin[1], Tad Ogorzalek[1], Paul D. Adams[1], Nathan Hillson[1], Christopher J Petzold[1]

[1]Lawrence Berkeley National Laboratory

1

dx.doi.org/10.17504/protocols.io.b3gxqjxn

LBNL omics | Agile BioFoundry | 1

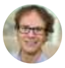

Christopher Petzold
Lawrence Berkeley National Laboratory

These protocols are for research purposes only.

Manual proteomic sample preparation methods limit sample throughput and often lead to poor data quality when thousands of samples must be analyzed. Automated workflows are increasingly used to overcome these issues for some (or even all) of the sample preparation steps. Here, we detail three optimised step-by-step protocols to: (A) lyse Gram-negative bacteria and fungal cells; (B) quantify the amount of protein extracted; and (C) normalize the amount of protein and set up tryptic digestion. These protocols have been developed to facilitate rapid, low variance sample preparation of hundreds of samples, be easily implemented on widely-available Beckman-Coulter Biomek automated liquid handlers, and allow flexibility for future protocol development. By using this workflow 50 micrograms of peptides for 96 samples can be prepared for tryptic digestion in under an hour. We validate these protocols by analyzing 47 E. coli and R. toruloides samples and show that this modular workflow provides robust, reproducible proteomic samples for high-throughput applications. The expected results from these protocols are 94 peptide samples from Gram-negative bacterial and fungal cells prepared for bottom-up quantitative proteomic analysis without the need for desalting column cleanup and with peptide variance (CVs) below 15%.

DOI

dx.doi.org/10.17504/protocols.io.b3gxqjxn

Citation: Yan Chen, Nurgul Kaplan Lease, Jennifer Gin, Tad Ogorzalek, Paul D. Adams, Nathan Hillson, Christopher J Petzold Modular automated bottom-up proteomic sample preparation for high-throughput applications https://dx.doi.org/10.17504/protocols.io.b3gxqjxn

This is an open access protocol distributed under the terms of the **Creative Commons Attribution License**

Yan Chen, Nurgul Kaplan Lease, Jennifer Gin, Tad Ogorzalek, Paul D. Adams, Nathan Hillson, Christopher J Petzold 2022. Modular automated bottom-up proteomic sample preparation for high-throughput applications. **protocols.io**
https://dx.doi.org/10.17504/protocols.io.b3gxqjxn
Christopher Petzold

Automation, Sample preparation, Proteomics, Protein quantification, Biomek, Lowry assay, Cell lysis, Normalization

collection ,

Jan 04, 2022

Jan 12, 2022

Jan 12, 2022 Christopher Petzold Lawrence Berkeley National Laboratory

56567

- A Beckman-Coulter Biomek FX liquid handler system with a 96-pod head is used for the protein extraction and quantification protocols. Alternative liquid handlers can be used with appropriate method development.

- A Beckman-Coulter Biomek NX-S8 or NXP liquid handler system with an 8-pod head is used for the normalization protocol. Alternative liquid handlers can be used with appropriate method development.

- A Molecular Devices Spectramax 250 microplate reader is used for the protein quantification assay measurement.

Notes:
- This protocol is set up to measure the amount of protein in duplicate.

For this protocol you will need:
- a Beckman-Coulter Biomek FX liquid handler system with a 96-pod head or similar liquid handler system
- a Beckman-Coulter Biomek NX-S8 or NXP liquid handler system with an 8-pod head or similar liquid handler system
- an Eppendorf 5810R centrifuge with S-4-104 rotor or similar centrifuge

Wear proper PPE (gloves, safety goggles, and lab coat), and prepare solvents in a chemical fume hood.

Citation: Yan Chen, Nurgul Kaplan Lease, Jennifer Gin, Tad Ogorzalek, Paul D. Adams, Nathan Hillson, Christopher J Petzold Modular automated bottom-up proteomic sample preparation for high-throughput applications
https://dx.doi.org/10.17504/protocols.io.b3gxqjxn

This is an open access protocol distributed under the terms of the **Creative Commons Attribution License**

Store organic solvents in a flammable storage cabinet when not in use.
Discard used solvents and buffers in appropriate waste containers.

DISCLAIMER:

These protocols are for research purposes only.

Manual proteomic sample preparation methods limit sample throughput and often lead to poor data quality when thousands of samples must be analyzed. Automated workflows are increasingly used to overcome these issues for some (or even all) of the sample preparation steps. Here, we detail three optimised step-by-step protocols to: (A) lyse Gram-negative bacteria and fungal cells; (B) quantify the amount of protein extracted; and (C) normalize the amount of protein and set up tryptic digestion. These protocols have been developed to facilitate rapid, low variance sample preparation of hundreds of samples, be easily implemented on widely-available Beckman-Coulter Biomek automated liquid handlers, and allow flexibility for future protocol development. By using this workflow 50 micrograms of peptides for 96 samples can be prepared for tryptic digestion in under an hour. We validate these protocols by analyzing 47 E. coli and R. toruloides samples and show that this modular workflow provides robust, reproducible proteomic samples for high-throughput applications. The expected results from these protocols are 94 peptide samples from Gram-negative bacterial and fungal cells prepared for bottom-up quantitative proteomic analysis without the need for desalting column cleanup and with peptide variance (CVs) below 15%.

FILES

Automated Chloroform-Methanol Protein Extraction on the Biomek-FX Liquid Handler System
Version 2
by Jennifer Gin, Lawrence Berkeley National Laboratory

Automated Protein Quantification with the Biomek-FX Liquid Handler System
Version 2
by Jennifer Gin, Lawrence Berkeley National Laboratory

Automated Protein Normalization and Tryptic Digestion on a Biomek-NX Liquid Handler System
Version 2
by Christopher Petzold, Lawrence Berkeley National Laboratory

Citation: Yan Chen, Nurgul Kaplan Lease, Jennifer Gin, Tad Ogorzalek, Paul D. Adams, Nathan Hillson, Christopher J Petzold Modular automated bottom-up proteomic sample preparation for high-throughput applications
https://dx.doi.org/10.17504/protocols.io.b3gxqjxn

This is an open access protocol distributed under the terms of the **Creative Commons Attribution License**

Citation: Yan Chen, Nurgul Kaplan Lease, Jennifer Gin, Tad Ogorzalek, Paul D. Adams, Nathan Hillson, Christopher J Petzold Modular automated bottom-up proteomic sample preparation for high-throughput applications
https://dx.doi.org/10.17504/protocols.io.b3gxqjxn

This is an open access protocol distributed under the terms of the **Creative Commons Attribution License**
