# Supplementary material for: Modular automated bottom-up proteomic sample preparation for high-throughput applications
Source: PLoS One. 2022 Feb 25;17(2):e0264467. doi: 10.1371/journal.pone.0264467 (PMC8880914; doi:10.1371/journal.pone.0264467)
Supplement: S2 File — Also available on protocols.io. (PDF) [file pone.0264467.s002.pdf]

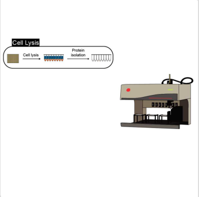

Automated Chloroform-Methanol Protein Extraction on the Biomek-FX Liquid Handler System

Automated Chloroform-Methanol Protein Extraction on the Biomek-FX Liquid Handler System

Yan Chen[1], Tad Ogorzalek[1], Nurgul Kaplan Lease[1], Jennifer Gin[1], Christopher J Petzold[1]

[1]Lawrence Berkeley National Laboratory

1

Christopher Petzold
Lawrence Berkeley National Laboratory

This protocol details steps to extract protein from Gram-negative bacterial or fungal cells (that have been pretreated with zymolyase) in quantitative proteomic workflows by using a Biomek FX liquid handler system. It is a semi-automated protocol that includes several 'pause' steps for centrifugation steps that are conducted manually "off-deck".

This protocol works best as part of an automated proteomic sample preparation workflow with:

Automated Protein Quantitation with the Biomek-FX liquid handler system

and

Automated Protein Normalization and Tryptic Digestion on a Biomek-NX Liquid Handler System

Yan Chen, Tad Ogorzalek, Nurgul Kaplan Lease, Jennifer Gin, Christopher J Petzold . Automated Chloroform-Methanol Protein Extraction on the Biomek-FX Liquid Handler System. **protocols.io**
https://protocols.io/view/automated-chloroform-methanol-protein-extraction-o-b3ryqm7w

Automated Chloroform-Methanol Protein Extraction on the Biomek-FX Liquid Handler System, Jennifer Gin

Cell lysis, Automation, Proteomics, Biomek, Sample preparation, Bacteria

Jan 12, 2022

Jan 12, 2022

56856

- A Beckman-Coulter Biomek FX liquid handler system is used for this protocol. Alternative liquid handlers can be used with appropriate method development.

- Because different deck orientations and system components are possible, you will need to modify the method file (attached in the 'Before start' section) for your specific Biomek liquid handler system.

96-well deep well plate Sigma Aldrich, Catalog #CLS3961-100EA
Hard-Shell 96-Well PCR Plates low profile thin wall skirted white/clearBIO-RADCatalog #HSP9601
200 uL pipet tips Molecular Bioproducts BioRobotix, Catalog #919-262
96 Deep Well Reagent Reservoir VWR, Catalog #101100-962
Methanol LC-MS grade B&J BrandVWR ScientificCatalog #BJLC230-2.5
Chloroform for HPLCSigma – AldrichCatalog #34854
Water LC-MS grade B&J BrandVWR ScientificCatalog #BJLC365-2.5
Ammonium Bicarbonate LC-MS gradeVWR ScientificCatalog #BJ40867-50G

Wear proper PPE (gloves, safety goggles, and lab coat), and prepare solvents in a chemical fume hood.
Store organic solvents in a flammable storage cabinet when not in use.
Discard used solvents and buffers in appropriate waste containers.

For this protocol you will need:
- a Beckman-Coulter Biomek FX liquid handler system with a 96-pod head
- Upload the attached method file and modify it to fit your deck and system configuration

Modular Protein Extraction method.bmf

- an Eppendorf 5810R centrifuge with S-4-104 rotor or similar centrifuge

Deck Setup 10m

1 Open Biomek Software that controls Biomek-FX liquid handler system. Under "File" drop-down, click "Open" to select the automation method "Modular Protein Extraction method."

> Because different deck orientations and system components are possible, you will need to modify the method file (attached in the 'Before start' section) for your specific Biomek liquid handler system.

2 Click on "Instrument Setup" under the "Setup" group node to get visual instruction on how to set up the deck.

3 Set up the deck (refer to the deck setup picture below):

10m

A	B	C
**Deck Label**	**Labware**	**Reagent**
**cells**	96-Well Deep Well Plate (Sigma Aldrich, Cat.#CLS3961-100EA)	4 OD units of cells
**prot**	PCR96 plate (BIO-RAD, Cat.#HSP9601)	
**tips1-5**	200 uL pipet tips (Molecular Bioproducts BioRobotix, Cat.#919-262)	
**water**	96 Deep Well Reagent Reservoir (VWR, Cat.#101100-962)	LC-MS grade Water (VWR Scientific, Cat.#BJLC365-2.5)
**solvent**	96 Deep Well Reagent Reservoir (VWR, Cat.#101100-962)	4:1 Methanol:Chloroform<br>LC-MS grade Methanol (VWR Scientific, Cat.#BJLC230-2.5), Chloroform (Sigma-Aldrich, Cat.#34854)
**buffer**	96 Deep Well Reagent Reservoir (VWR, Cat.#101100-962)	100 milimolar (mM) Ammonium Bicarbonate in 20% Methanol
**MeOH**	96 Deep Well Reagent Reservoir (VWR, Cat.#101100-962)	LC-MS grade Methanol (VWR Scientific, Cat.#BJLC230-2.5)

Materials for Deck setup
Note: 1 OD unit = 1 ml of cell culture at OD600 measurement of 1.0

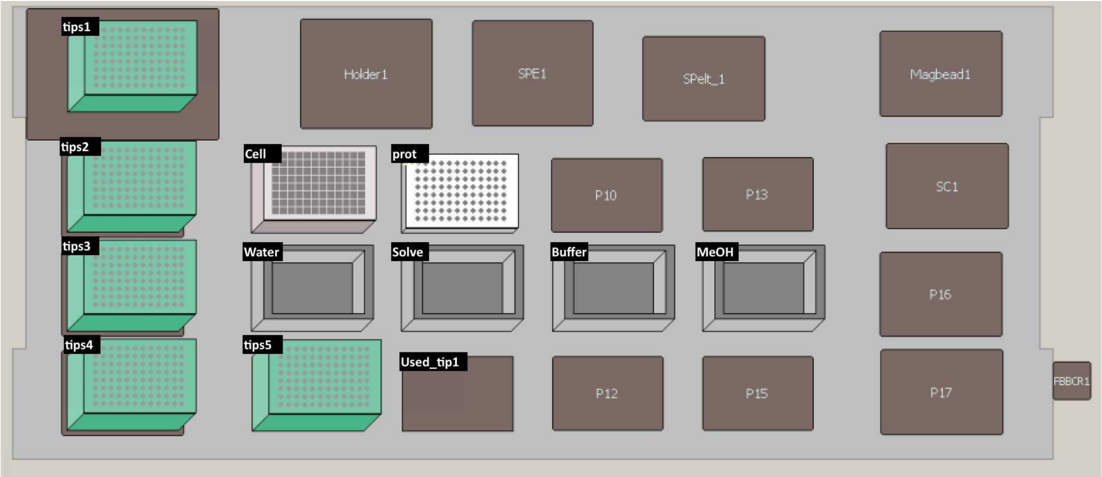

Deck Setup

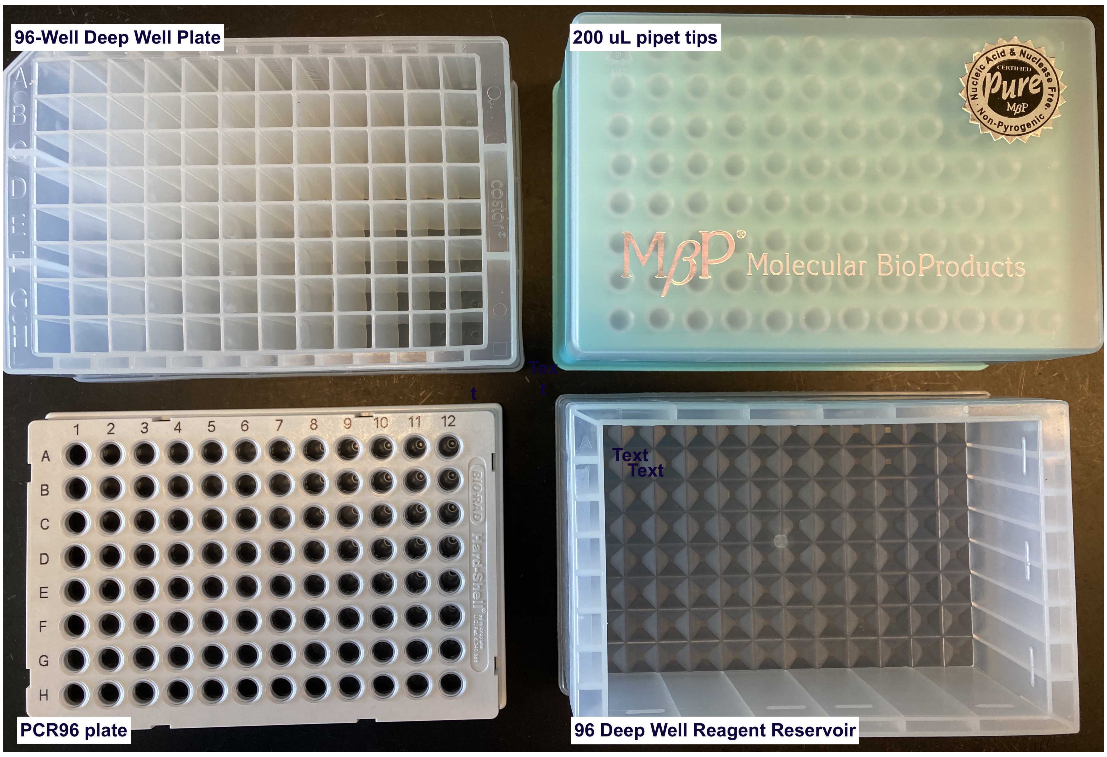

Labware for Deck setup

4 Click the "Run" button (green arrow) to start.

Protein Extraction 20m

5 Transfer 87.5 µl of water from water reservoir to cell plate. Mix and resuspend cell pellet with 20 cycles of pipetting mixing on deck. 2m

Note: The number of pipetting cycles is a user defined variable in the Biomek method that can be changed as needed.

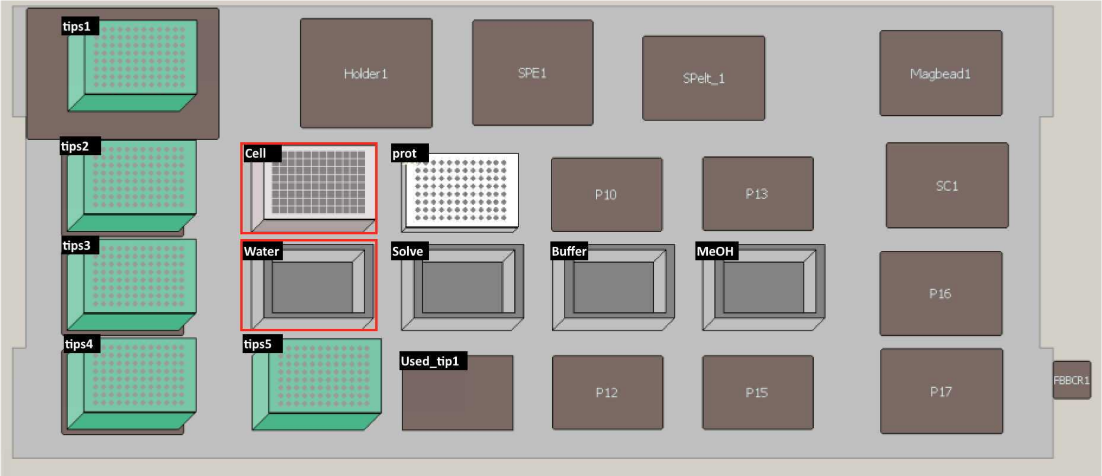

6 Transfer cell resuspension to PCR96 plate. 1m

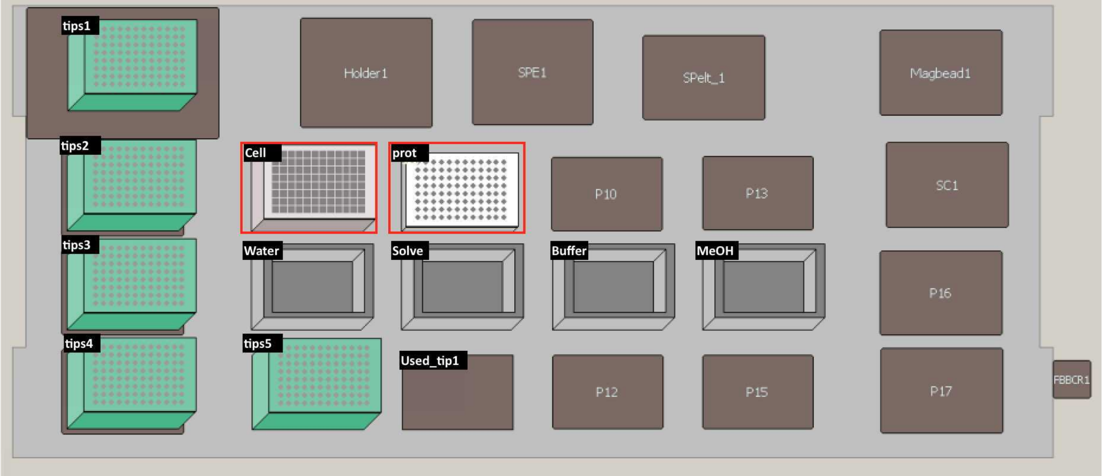

7

The PAUSE step prompts you to centrifuge your PCR96 plate.

8 Centrifuge **2000 x g, 25°C, 00:02:00** . 2m

9 Put PCR96 plate back on deck space (refer to deck setup picture above) -- PCR96 plate "prot."
Swirl and pour solvent (4:1, Methanol:Chloroform) into reservoir.

> Note: It is important to swirl the Methanol:Chloroform mixture at this step to ensure it is well mixed.

10 Remove supernatant by transferring 87 µl from PCR96 plate back to cell plate. 2m

11 Transfer 125 µl of solvent (4:1, Methanol:Chloroform) to PCR96 plate. Mix to promote cell lysis with 20 cycles of pipetting mixing on deck. 2m

Note: The number of pipetting cycle is user defined variable that could be changed as needed.

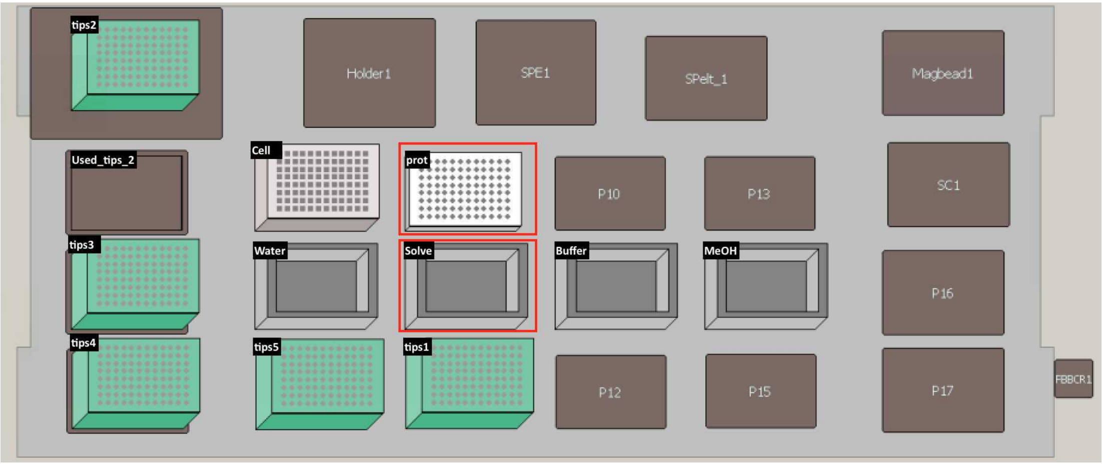

12 Transfer 75 µl of water into PCR96 plate and seal the plate with peelable seal at microplate sealer. Gently mix the samples by inverting the plate 5 times. 1m

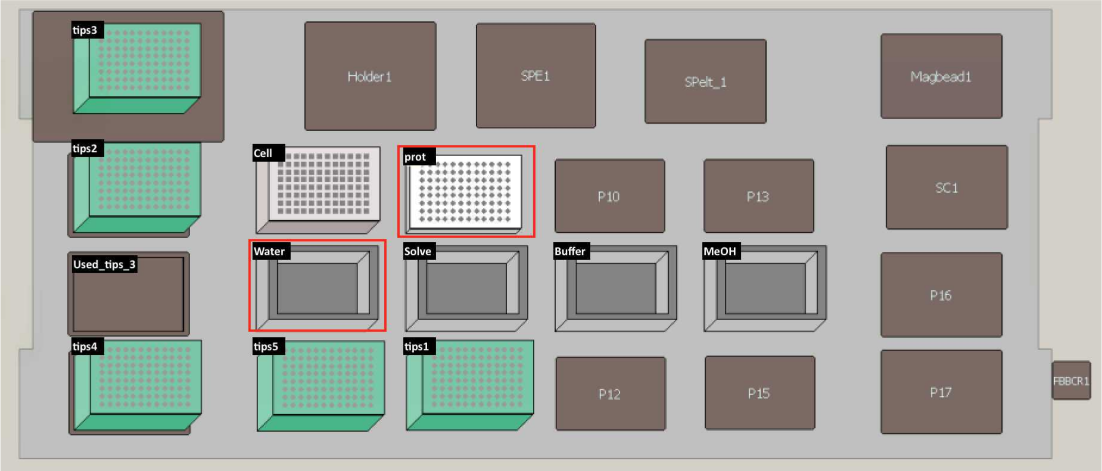

13 The PAUSE step prompts you to centrifuge your PCR96 plate.

14 Centrifuge **4000 rpm, 25°C, 00:02:00** . 2m

Visualize your plate after centrifugation to ensure that protein forms a nice pellet layer in the middle. Add centrifugation time as needed.

15 Put PCR96 plate back on deck space (refer to deck setup picture above) -- PCR96 plate "prot."

16 Remove supernatant by transferring 170 µl of top layer from PCR96 plate to cell plate. 1m

17 Add 75 µl of methanol and mix with 20 cycles of pipetting mixing on deck. 3m

Note: The number of pipetting cycles is a user defined variable in the Biomek method that can be changed as needed.

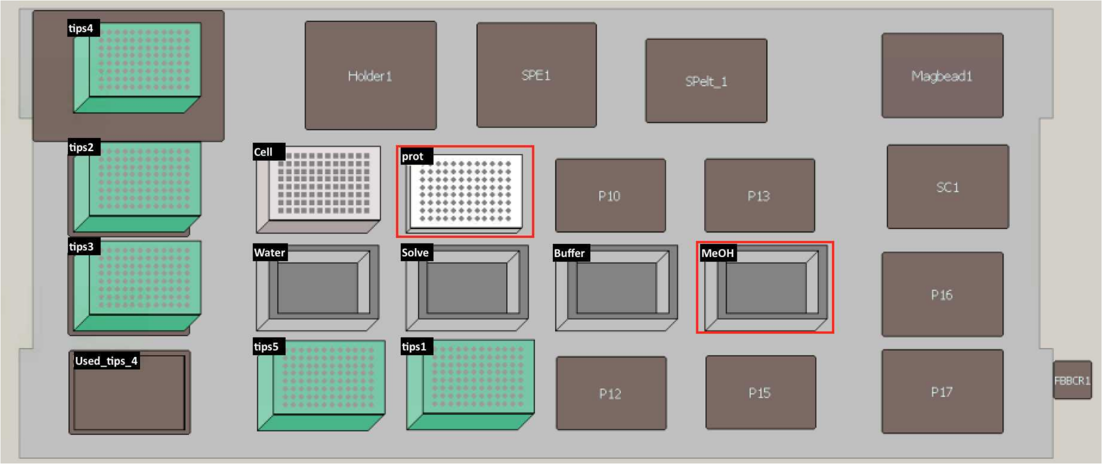

18 The PAUSE step prompts you to centrifuge your PCR96 plate.

Before you centrifuge, visualize your plate to make sure the Chloroform layer has mixed well with added Methanol.

19 Centrifuge **4000 rpm, 25°C, 00:02:00** . 2m

20 Put PCR96 plate back on deck space (refer to deck setup picture above) -- PCR96 plate "prot."

21 Remove supernatant. 1m

22 Resuspend in **100 µl** 1m

[M]100 Milimolar (mM) Ammonium Bicarbonate in 20% Methanol and mix the samples with user defined cycles of pipetting mixing on deck.

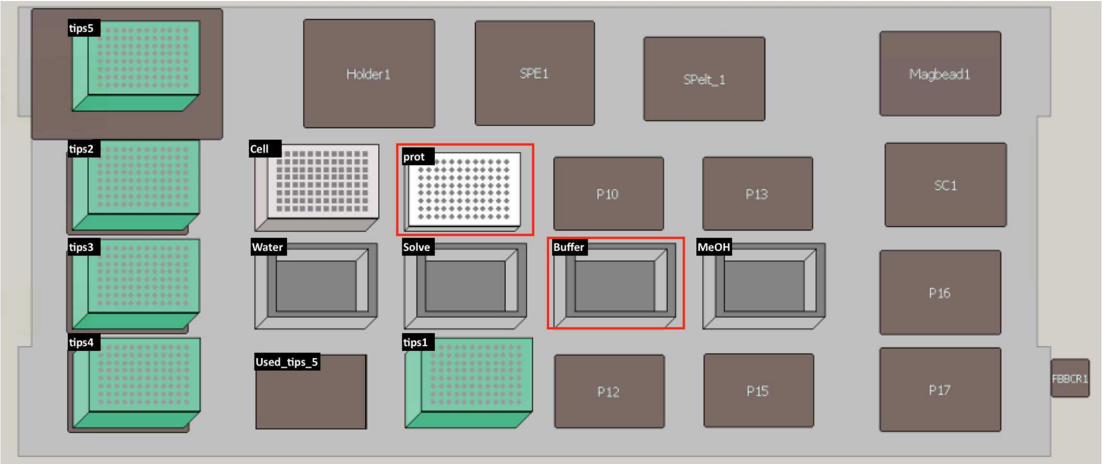

Note: 1. Samples are typically cloudy in this step. After trypsin digestion they will be nearly clear.
2. Inspecting samples after method finishes in case chunks of protein may present in samples of large protein pellets, Mixing with multichannel pipette may be necessary.
3. Users can increase or decrease resuspension volume as needed.

23 Store at **-20 °C** until ready for [Automated Protein Quantitation with the Biomek-FX liquid handler system]().
