# Supplementary material for: Modular automated bottom-up proteomic sample preparation for high-throughput applications
Source: PLoS One. 2022 Feb 25;17(2):e0264467. doi: 10.1371/journal.pone.0264467 (PMC8880914; doi:10.1371/journal.pone.0264467)
Supplement: S3 File — Also available on protocols.io. (PDF) [file pone.0264467.s003.pdf]

OPEN ACCESS

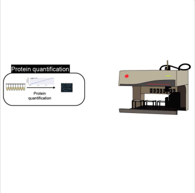

2

Jan 12, 2022

Automated Protein Quantification with the Biomek-FX Liquid Handler System V.2

1

Yan Chen[1], Nurgul Kaplan Lease[1], Tad Ogorzalek[1], Jennifer Gin[1], Christopher J Petzold[1]

[1]Lawrence Berkeley National Laboratory

1

dx.doi.org/10.17504/protocols.io.b3grqjv6

LBNL omics Agile BioFoundry 1

Jennifer Gin
Lawrence Berkeley National Laboratory

This protocol details steps to perform the protein quantification (Lowry-based) assay by using a Biomek FX liquid handler system. It is optimized to assay a full 96-well plate of protein samples in duplicate with a separate (control) plate for BSA standards. You will need a plate reader to measure the samples and standards.

This protocol works best as part of a full proteomic sample preparation workflow with:

Automated Chloroform-Methanol Protein Extraction on the Biomek-FX Liquid Handler System

and

Automated Protein Normalization and Tryptic Digestion on a Biomek-FX Liquid Handler System

DOI

dx.doi.org/10.17504/protocols.io.b3grqjv6

Yan Chen, Nurgul Kaplan Lease, Tad Ogorzalek, Jennifer Gin, Christopher J Petzold 2022. Automated Protein Quantification with the Biomek-FX Liquid Handler System. **protocols.io**
https://dx.doi.org/10.17504/protocols.io.b3grqjv6
Christopher Petzold

Citation: Yan Chen, Nurgul Kaplan Lease, Tad Ogorzalek, Jennifer Gin, Christopher J Petzold Automated Protein Quantification with the Biomek-FX Liquid Handler System https://dx.doi.org/10.17504/protocols.io.b3grqjv6

This is an open access protocol distributed under the terms of the **Creative Commons Attribution License** (https://creativecommons.org/licenses/by/4.0/), which permits unrestricted use, distribution, and reproduction in any medium,

Modular automated bottom-up proteomic sample preparation for high-throughput applications

Protein quantification, Automation, Biomek, Lowry assay, Proteomics, Sample preparation

protocol ,

Jonathan Vu

Jan 04, 2022

Jan 12, 2022

Jan 12, 2022 Jennifer Gin Lawrence Berkeley National Laboratory

56561

Part of collection

Modular automated bottom-up proteomic sample preparation for high-throughput applications

- A Beckman-Coulter Biomek FX liquid handler system with a 96-pod head is used for this protocol. Alternative liquid handlers can be used with appropriate method development.

- A Molecular Devices Spectramax 250 microplate reader is used for the protein quantification assay measurement.

- Because different deck orientations and system components are possible, you will need to modify the method file (attached in the 'Before start' section) for your specific Biomek liquid handler system.

Notes:
- This protocol is set up to measure the amount of protein in duplicate.

Citation: Yan Chen, Nurgul Kaplan Lease, Tad Ogorzalek, Jennifer Gin, Christopher J Petzold Automated Protein Quantification with the Biomek-FX Liquid Handler System https://dx.doi.org/10.17504/protocols.io.b3grqjv6

This is an open access protocol distributed under the terms of the **Creative Commons Attribution License** (https://creativecommons.org/licenses/by/4.0/), which permits unrestricted use, distribution, and reproduction in any medium,

Hard-Shell 96-Well PCR Plates low profile thin wall skirted white/clearBIO-RADCatalog #HSP9601
Pierce Bovine Serum Albumin Standard Pre-Diluted Set Thermo Fisher, Catalog #23208
20 uL pipet tips Molecular Bioproducts BioRobotix, Catalog #918-262
200 uL pipet tips Molecular Bioproducts BioRobotix, Catalog #919-262
Corning 96 Well Black Polystyrene Microplate Fisher Scientific, Catalog #07-200-567
Reservoir Microplate Agilent, Catalog #201254-100
96 Deep Well Reagent Reservoir VWR, Catalog #101100-962
Water LC-MS grade B&J Brand VWR Scientific, Catalog #BJLC365-2.5
DC Protein Assay Reagent A by Bio-rad Laboratories, Catalog #500-0113
DC Protein Assay Reagent B by Bio-rad Laboratories, Catalog #500-0114
8-strip PCR Tubes with Caps Axygen, Catalog #14-222-251

Wear proper PPE (gloves, safety goggles, and lab coat), and prepare solvents in a chemical fume hood.
Store organic solvents in a flammable storage cabinet when not in use.
Discard used solvents and buffers in appropriate waste containers.

Prepare BSA Standards Plate (1st 4 rows from A to D):
1. Add 40 uL of H_2O into wells A1 to D1.
2. Add 40 uL of BSA Standards 1 (125 ug/mL) to 7 (2000 ug/mL) into columns 2 to 8.

For this protocol you will need:
1. Beckman-Coulter Biomek FX liquid handler system with a 96-pod head.
2. Upload the attached method file and modify it to fit your deck and system configuration.
   Modular Protein Quantitation method.bmf

Deck Setup 10m

1 Open Biomek Software that controls Biomek-FX liquid handler system. Under "File" drop-down menu, click "Open" to select the automation method "Modular Protein Quantitation method."

> Because different deck orientations and system components are possible, you will need to modify the method file (attached in the 'Before start' section) for your specific Biomek liquid handler system.

2 Click on "Instrument Setup" under the "Setup" group node to get visual instruction on how to set up the deck.

3 Set up the deck (refer to the deck setup picture below): 10m

Citation: Yan Chen, Nurgul Kaplan Lease, Tad Ogorzalek, Jennifer Gin, Christopher J Petzold Automated Protein Quantification with the Biomek-FX Liquid Handler System https://dx.doi.org/10.17504/protocols.io.b3grqjv6

This is an open access protocol distributed under the terms of the **Creative Commons Attribution License** (https://creativecommons.org/licenses/by/4.0/), which permits unrestricted use, distribution, and reproduction in any medium,

A	B	C
**Deck Label**	**Labware**	**Reagent**
**protein**	PCR96 plate (BIO-RAD, Cat.#HSP9601)	unknown amount of protein to quantify
**titrate**	PCR96 plate (BIO-RAD, Cat.#HSP9601)	
**BSA standards**	PCR96 plate	BSA Standards (Thermo Fisher, Cat.#23208)
**tips1,2**	20 µl pipet tips (Molecular Bioproducts BioRobotix, Cat.#918-262)	
**tips 3,5**	200 uL pipet tips (Molecular Bioproducts BioRobotix, Cat.#919-262)	
**control read, prot read 1, prot read 2**	Black Microplate (Fisher Scientific, Cat.#07-200-567)	
**Buffer A**	Reservoir Microplate (Agilent, Cat.#201254-100)	DC Protein Assay Reagent A (Bio-rad Laboratories, Cat.#500-0113)
**water**	Reservoir Microplate (Agilent, Cat.#201254-100)	LC-MS grade Water (VWR Scientific, Cat.#BJLC365-2.5)
**Buffer B**	96 Deep Well Reagent Reservoir (VWR, Cat.#101100-962)	DC Protein Assay Reagent B (Bio-rad Laboratories, Cat.#500-0114)

Materials for Deck setup

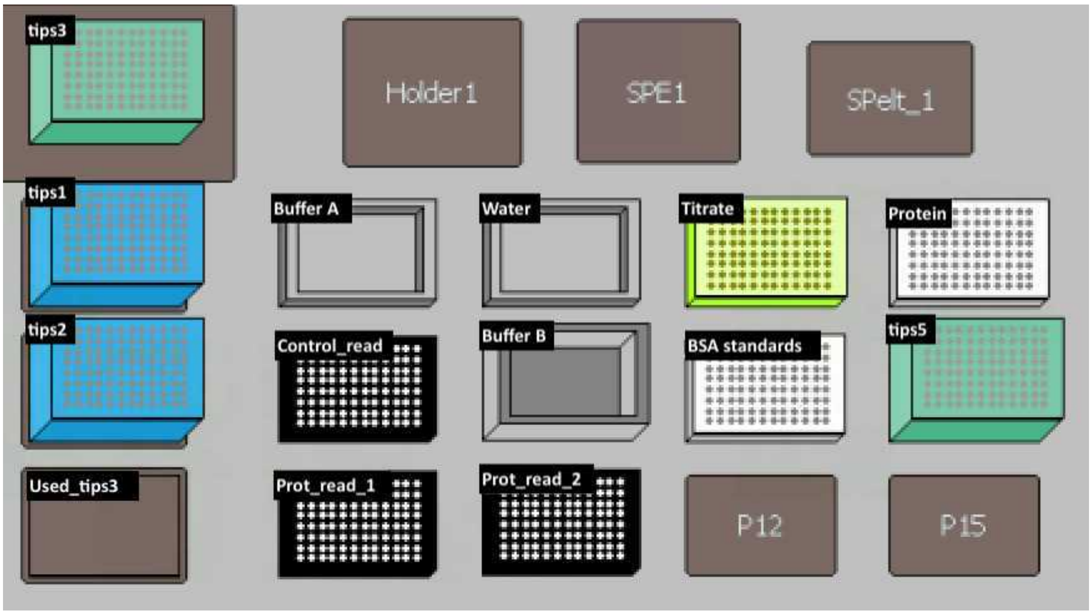

Deck setup

Citation: Yan Chen, Nurgul Kaplan Lease, Tad Ogorzalek, Jennifer Gin, Christopher J Petzold Automated Protein Quantification with the Biomek-FX Liquid Handler System https://dx.doi.org/10.17504/protocols.io.b3grqjv6

This is an open access protocol distributed under the terms of the **Creative Commons Attribution License** (https://creativecommons.org/licenses/by/4.0/), which permits unrestricted use, distribution, and reproduction in any medium,

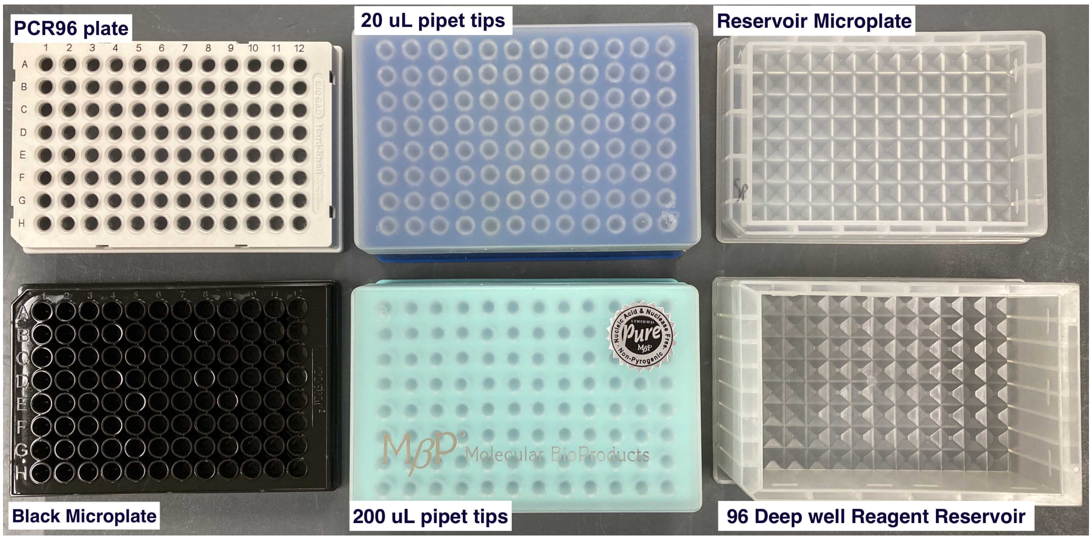

Labware for Deck setup

4 MANUAL STEP: Use a multichannel pipette to mix protein samples right before starting. 2m

5 Click the "Run" button (green arrow) to start.

DC protein assay 25m

6 Transfer 25 µl of Buffer A to Protein Read plate 1, Protein Read plate 2, and Control Read plate. 1m

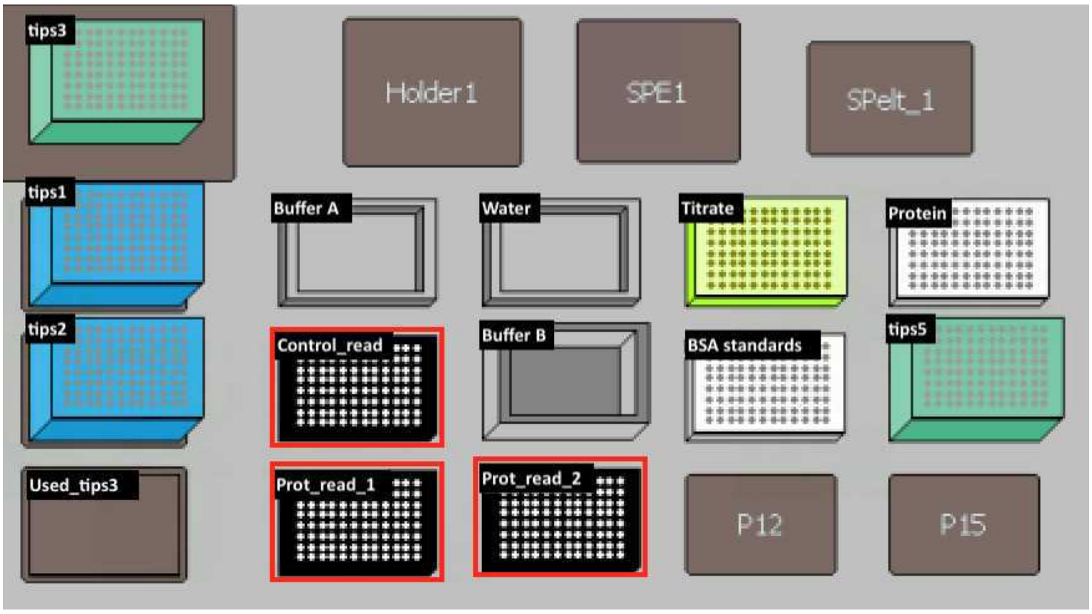

Citation: Yan Chen, Nurgul Kaplan Lease, Tad Ogorzalek, Jennifer Gin, Christopher J Petzold Automated Protein Quantification with the Biomek-FX Liquid Handler System https://dx.doi.org/10.17504/protocols.io.b3grqjv6

This is an open access protocol distributed under the terms of the **Creative Commons Attribution License** (https://creativecommons.org/licenses/by/4.0/), which permits unrestricted use, distribution, and reproduction in any medium,

7 Transfer 12 µl of H_2O into Titrate plate. Then transfer 3 µl (see Note for more details) from Protein plate to Titrate plate and mix with 5 cycles of pipetting mixing on deck. 1m

Note: The dilution factor could be altered as needed by changing the volumes of water and proteins transferred to the titration plate. Be sure to multiply the protein quant results by the dilution factor before you do your normalization calculation.

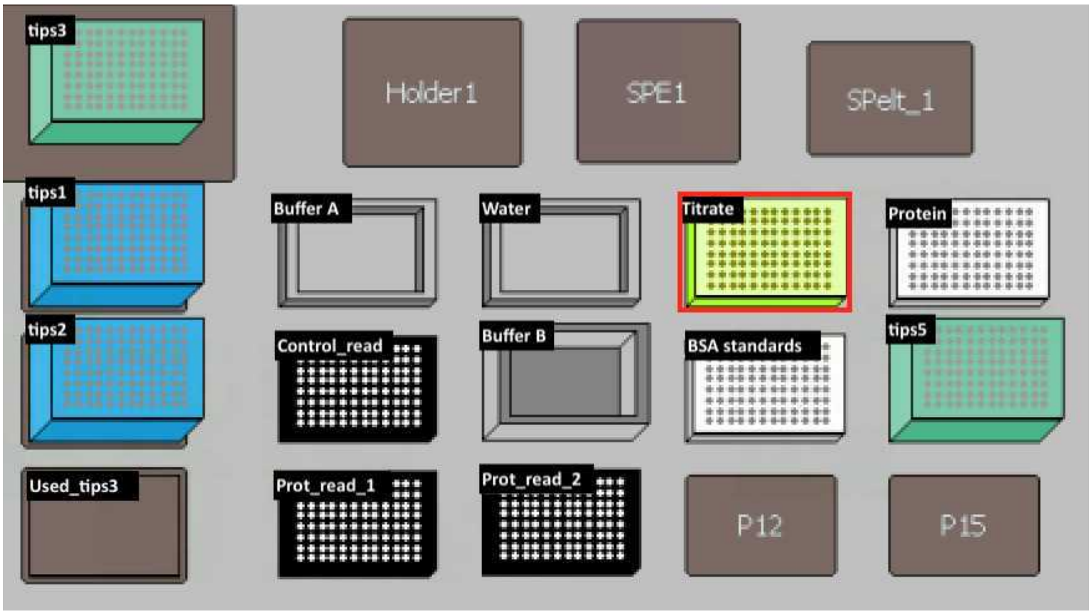

8 Transfer 5 µl of protein from Titrate plate to Protein Read plate 1 and Protein Read plate 2. 1m

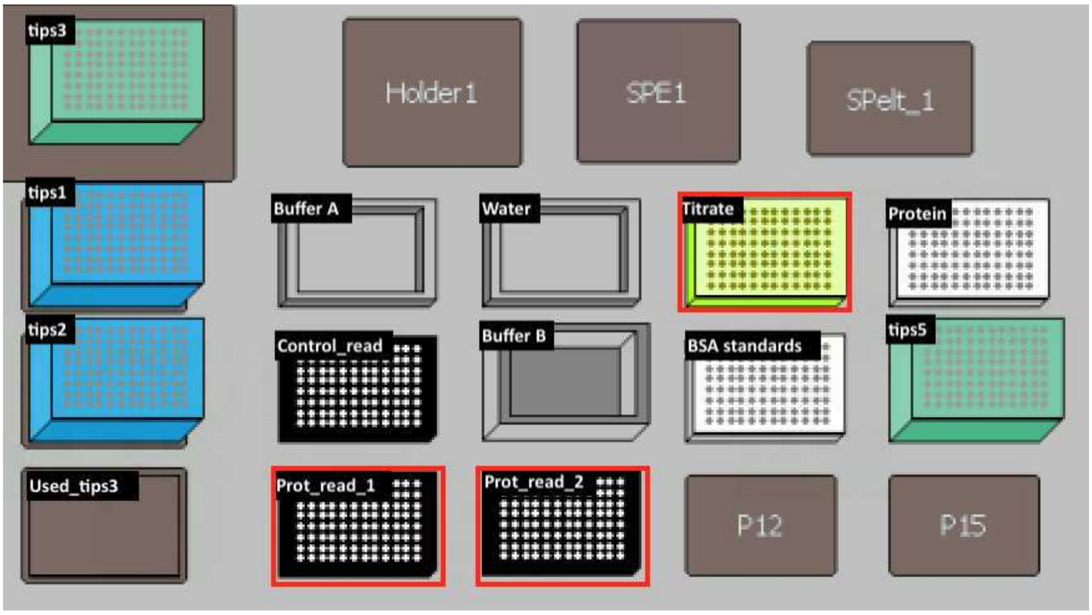

Citation: Yan Chen, Nurgul Kaplan Lease, Tad Ogorzalek, Jennifer Gin, Christopher J Petzold Automated Protein Quantification with the Biomek-FX Liquid Handler System https://dx.doi.org/10.17504/protocols.io.b3grqjv6

This is an open access protocol distributed under the terms of the **Creative Commons Attribution License** (https://creativecommons.org/licenses/by/4.0/), which permits unrestricted use, distribution, and reproduction in any medium,

9 Transfer 5 µl from BSA Standards plate to Control Read plate. 1m

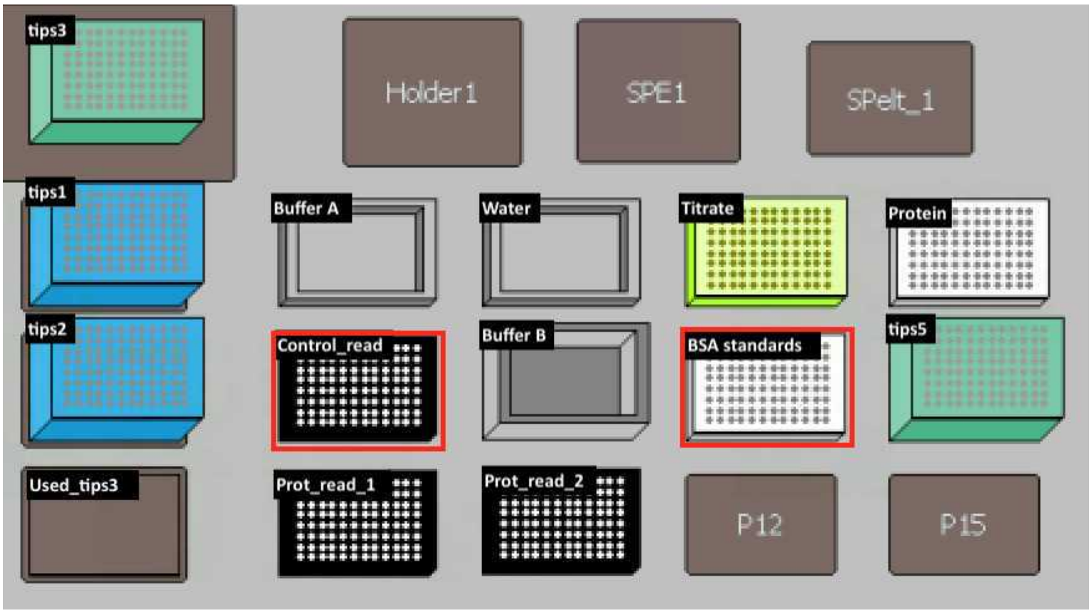

Prepare BSA Standards plate (1st 4 rows from A to D):
Add 40 µl of H_2O into wells A1 to D1.
Add 40 µl of BSA Standards 1 (125 µg/ml) to 7 (2000 µg/ml) into columns 2 to 8.

10 Method will pause until user resumes it again. Set a timer for 5 minutes. 5m

11 After 5 min, click OK to resume method.

12 Transfer 200 µl from Buffer B to the 3 Read plates and incubate for **00:10:00** . 10m

The method will pause until user resumes it again. Set up a 10 minutes timer and click OK afterwards to finish.

Citation: Yan Chen, Nurgul Kaplan Lease, Tad Ogorzalek, Jennifer Gin, Christopher J Petzold Automated Protein Quantification with the Biomek-FX Liquid Handler System https://dx.doi.org/10.17504/protocols.io.b3grqjv6

This is an open access protocol distributed under the terms of the **Creative Commons Attribution License** (https://creativecommons.org/licenses/by/4.0/), which permits unrestricted use, distribution, and reproduction in any medium,

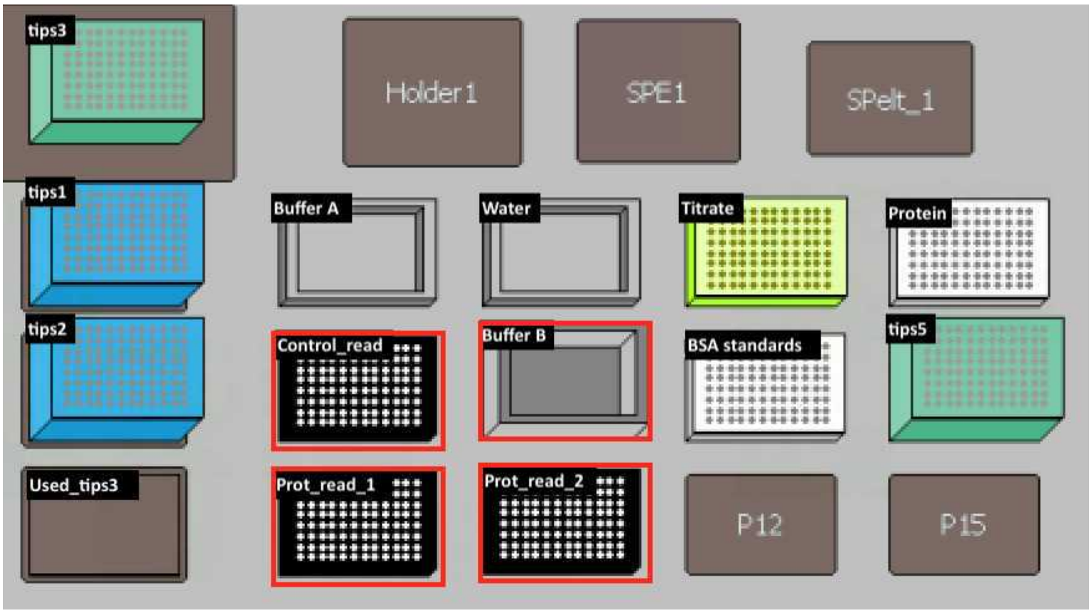

Spectrophotometer reading for protein Quantification 10m

13 Transfer plates to the microplate reader (MD Spectramax 250) to read absorbance at 750 nm and calculate protein concentration. 1m

14 Read Control Read plate. 1m

A	B	C
**Sample**	**Concentration**	**Mean OD Value (Absorbance)**
St01	125	0.024
St02	250	0.042
St03	500	0.090
St04	750	0.138
St05	1000	0.165
St06	1500	0.239
St07	2000	0.281

Standards (µg/ml)

Citation: Yan Chen, Nurgul Kaplan Lease, Tad Ogorzalek, Jennifer Gin, Christopher J Petzold Automated Protein Quantification with the Biomek-FX Liquid Handler System https://dx.doi.org/10.17504/protocols.io.b3grqjv6

This is an open access protocol distributed under the terms of the **Creative Commons Attribution License** (https://creativecommons.org/licenses/by/4.0/), which permits unrestricted use, distribution, and reproduction in any medium,

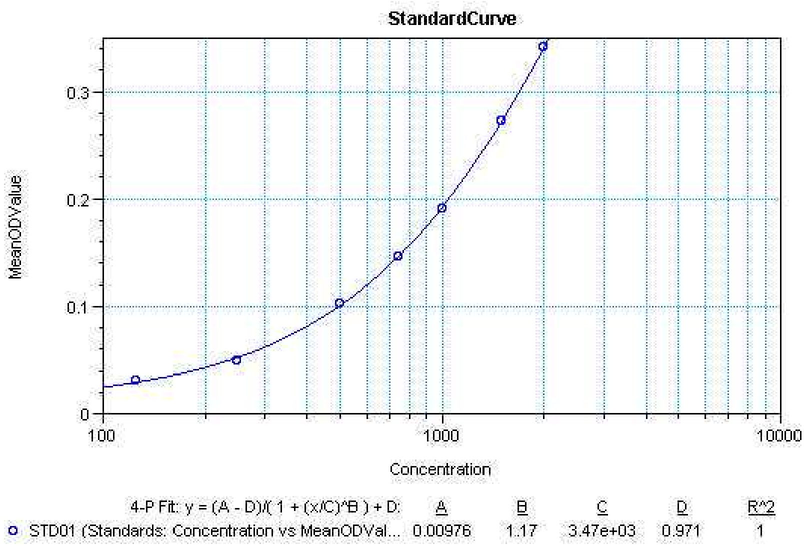

Example Standards Curve

15 Read Protein Read plate 1 and Protein Read plate 2.

2m

A	B
**Sample**	**Concentration**
Un88	585.249
Un89	785.257
Un90	670.135
Un91	718.864
Un92	868.962
Un93	679.907
Un94	743.064
Un95	994.173
Un96	1115.072

Examples of Sample concentrations that are reported by MD Spectramax 250

Remember to multiply the protein concentrations by five (5) to account for the five-fold dilution in Step #7.

Citation: Yan Chen, Nurgul Kaplan Lease, Tad Ogorzalek, Jennifer Gin, Christopher J Petzold Automated Protein Quantification with the Biomek-FX Liquid Handler System https://dx.doi.org/10.17504/protocols.io.b3grqjv6

This is an open access protocol distributed under the terms of the **Creative Commons Attribution License** (https://creativecommons.org/licenses/by/4.0/), which permits unrestricted use, distribution, and reproduction in any medium,

16 Store protein plate at -20 °C until ready for Automated Protein Normalization and Tryptic Digestion on a Biomek-NX Liquid Handler System.

Citation: Yan Chen, Nurgul Kaplan Lease, Tad Ogorzalek, Jennifer Gin, Christopher J Petzold Automated Protein Quantification with the Biomek-FX Liquid Handler System https://dx.doi.org/10.17504/protocols.io.b3grqjv6

This is an open access protocol distributed under the terms of the **Creative Commons Attribution License** (https://creativecommons.org/licenses/by/4.0/), which permits unrestricted use, distribution, and reproduction in any medium,
