# Supplementary material for: Modular automated bottom-up proteomic sample preparation for high-throughput applications
Source: PLoS One. 2022 Feb 25;17(2):e0264467. doi: 10.1371/journal.pone.0264467 (PMC8880914; doi:10.1371/journal.pone.0264467)
Supplement: S4 File — Also available on protocols.io. (PDF) [file pone.0264467.s004.pdf]

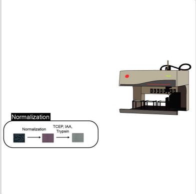


2
Jan 12, 2022

# Automated Protein Normalization and Tryptic Digestion on a Biomek-NX Liquid Handler System V.2

1

Yan Chen[1], Tad Ogorzalek[1], Nurgul Kaplan Lease[1], Jennifer Gin[1], Christopher J Petzold[1]

[1]Lawrence Berkeley National Laboratory

1

dx.doi.org/10.17504/protocols.io.b3gtqjwn

LBNL omics Agile BioFoundry 1

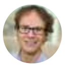

Christopher Petzold
Lawrence Berkeley National Laboratory

This protocol details steps to normalize the amount of protein for tryptic digestion in quantitative proteomic workflows by using a Biomek NX liquid handler system. It is optimized to normalize protein concentrations in a 96-well plate format and add TCEP, IAA, and trypsin.

This protocol works best as part of a semi-automated proteomic sample preparation workflow with:

Automated Chloroform-Methanol Protein Extraction on the Biomek-FX Liquid Handler System

and

Automated Protein Quantitation with the Biomek-FX liquid handler system

DOI

dx.doi.org/10.17504/protocols.io.b3gtqjwn

Yan Chen, Tad Ogorzalek, Nurgul Kaplan Lease, Jennifer Gin, Christopher J Petzold 2022. Automated Protein Normalization and Tryptic Digestion on a Biomek-NX Liquid Handler System. **protocols.io**
https://dx.doi.org/10.17504/protocols.io.b3gtqjwn
Yan Chen



**Citation:** Yan Chen, Tad Ogorzalek, Nurgul Kaplan Lease, Jennifer Gin, Christopher J Petzold Automated Protein Normalization and Tryptic Digestion on a Biomek-NX Liquid Handler System https://dx.doi.org/10.17504/protocols.io.b3gtqjwn

This is an open access protocol distributed under the terms of the **Creative Commons Attribution License** (https://creativecommons.org/licenses/by/4.0/), which permits unrestricted use, distribution, and reproduction in any medium,

ⓘ

## Modular automated bottom-up proteomic sample preparation for high-throughput applications

Automation, Proteomics, Tryptic digestion, Biomek, Normalization, Sample preparation

protocol ,

Jan 04, 2022

Jan 12, 2022

Jan 12, 2022 Christopher Petzold Lawrence Berkeley National Laboratory

56563

Part of collection

Modular automated bottom-up proteomic sample preparation for high-throughput applications

- A Beckman-Coulter Biomek NX-S8 or NXP liquid handler system with an 8-pod head is used for this protocol. Alternative liquid handlers can be used with appropriate method development.

- Because different deck orientations and system components are possible, you will need to modify the method file (attached in the 'Before start' section) for your specific Biomek liquid handler system.

PCR Plate 96-well non-skirted Thermo Fisher Scientific Catalog #AB0600
PCR Tube Storage Rack Axygen Catalog #R96PCRFSP
Ammonium Bicarbonate LC-MS grade VWR Scientific Catalog #BJ40867-50G
20 uL pipet tips Molecular Bioproducts BioRobotix, Catalog #918-262
200 uL pipet tips Molecular Bioproducts BioRobotix, Catalog #919-262
Tris(2-carboxyethyl)phosphine hydrochloride (TCEP), SigmaAldrich, Catalog #C4706
Iodoacetamide, MilliporeSigma, Catalog #I1149
Trypsin, SigmaAldrich, Catalog #T6567-1MG

Wear proper PPE (gloves, safety goggles, and lab coat), and prepare solvents in a chemical fume hood.
Store organic solvents in a flammable storage cabinet when not in use.
Discard used solvents and buffers in appropriate waste containers.

For this protocol you will need:



**Citation:** Yan Chen, Tad Ogorzalek, Nurgul Kaplan Lease, Jennifer Gin, Christopher J Petzold Automated Protein Normalization and Tryptic Digestion on a Biomek-NX Liquid Handler System https://dx.doi.org/10.17504/protocols.io.b3gtqjwn

This is an open access protocol distributed under the terms of the **Creative Commons Attribution License** (https://creativecommons.org/licenses/by/4.0/), which permits unrestricted use, distribution, and reproduction in any medium,

- A Beckman-Coulter Biomek NX-S8 or NXP liquid handler system with a 8-pod head
- Upload the attached method files and modify them to fit your deck and system configuration.

**Proteomics-Normalization Method.bmf**

**Proteomics-Plate to Plate Transfer.bmf**

- Protein samples of known concentration

Chemicals to prepare:

- Prepare [M]**100 Milimolar (mM) Tris(2-carboxyethyl)phosphine (TCEP) solution** by dissolving **28.7 mg TCEP** in **1 mL 100mM Ammonium Bicarbonate**
- Prepare [M]**200 Milimolar (mM) Iodoacetamide (IAA) solution** by dissolving **36.8 mg Iodoacetamide** in **1 mL 100mM Ammonium Bicarbonate**
- Prepare [M]**1 mg/ml Trypsin** by adding **1 mL 1mM HCl** to **1 mg Trypsin** then vortex to mix

Store TCEP, IAA, and Trypsin in -20C.
IAA is light sensitive. Store in amber tube (Fisher Scientific, Cat.#05-402-31).

## Biomek NX-S8 input file preparation

1 After measuring protein concentration by the DC (Detergent Compatible) protein assay (Bio-Rad), export protein concentration report through MD Spectramax 250 software that controls the microplate reader. Copy the content in the exported text file and paste it into Excel, and then save as a UTF-8 format text file.

**Example protein concentration file.txt**

2 Use MS Excel or a Jupyter notebook to normalize the protein concentration to **50 µg** and convert the spectrophotometer output file into the following two files in a format suitable for the Biomek NX-S8:

NX-AMBIC.csv



**Citation:** Yan Chen, Tad Ogorzalek, Nurgul Kaplan Lease, Jennifer Gin, Christopher J Petzold Automated Protein Normalization and Tryptic Digestion on a Biomek-NX Liquid Handler System https://dx.doi.org/10.17504/protocols.io.b3gtqjwn

This is an open access protocol distributed under the terms of the **Creative Commons Attribution License** (https://creativecommons.org/licenses/by/4.0/), which permits unrestricted use, distribution, and reproduction in any medium,

| A | B | C | D | E |
|---|---|---|---|---|
| **scrpos** | **srcwell** | **destpos** | **destwell** | **vol** |
| media | 1 | DestPlate | 1 | 33.05 |
| media | 1 | DestPlate | 2 | 26.73 |
| media | 1 | DestPlate | 3 | 27.96 |
| media | 1 | DestPlate | 4 | 28.74 |
| media | 1 | DestPlate | 5 | 22.94 |
| media | 1 | DestPlate | 6 | 28.07 |
| media | 1 | DestPlate | 7 | 24.34 |
| media | 1 | DestPlate | 8 | 28.12 |
| media | 1 | DestPlate | 9 | 26.64 |
| media | 1 | DestPlate | 10 | 26.22 |

NX-AMBIC.csv output table

NX-protein.csv

| A | B | C | D | E |
|---|---|---|---|---|
| **scrpos** | **srcwell** | **destpos** | **destwell** | **vol** |
| SrcPlate | 1 | DestPlate | 1 | 10.95 |
| SrcPlate | 2 | DestPlate | 2 | 17.27 |
| SrcPlate | 3 | DestPlate | 3 | 16.04 |
| SrcPlate | 4 | DestPlate | 4 | 15.26 |
| SrcPlate | 5 | DestPlate | 5 | 21.06 |
| SrcPlate | 6 | DestPlate | 6 | 15.93 |
| SrcPlate | 7 | DestPlate | 7 | 19.66 |
| SrcPlate | 8 | DestPlate | 8 | 15.88 |
| SrcPlate | 9 | DestPlate | 9 | 17.36 |
| SrcPlate | 10 | DestPlate | 10 | 17.78 |

NX-protein.csv output table

## Biomek NX-S8 preparation

3 Open Biomek software program from Biomek NX-S8 control computer. In the "Instrument" drop-down menu, select "Home all Axes" to prepare the instrument for use and purge air from the tubing and syringes.



**Citation:** Yan Chen, Tad Ogorzalek, Nurgul Kaplan Lease, Jennifer Gin, Christopher J Petzold Automated Protein Normalization and Tryptic Digestion on a Biomek-NX Liquid Handler System https://dx.doi.org/10.17504/protocols.io.b3gtqjwn

This is an open access protocol distributed under the terms of the **Creative Commons Attribution License** (https://creativecommons.org/licenses/by/4.0/), which permits unrestricted use, distribution, and reproduction in any medium,

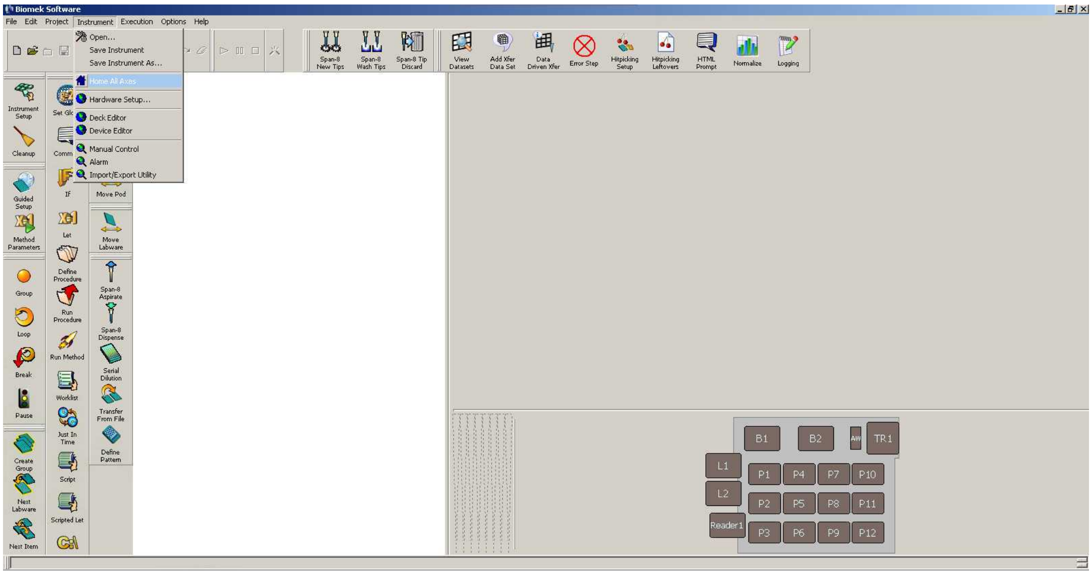


Biomek software that controls the operation of Biomek NX-S8 liquid handler system

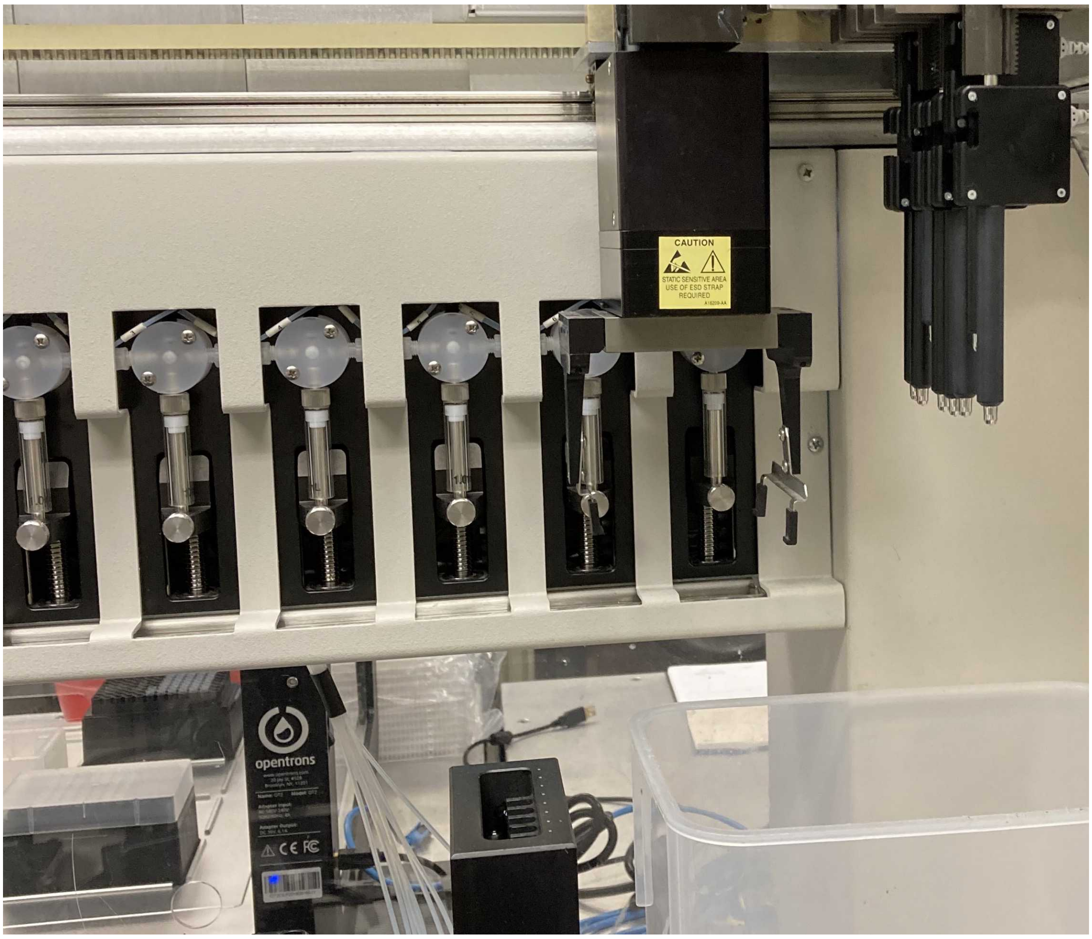


Homing all axes



**Citation:** Yan Chen, Tad Ogorzalek, Nurgul Kaplan Lease, Jennifer Gin, Christopher J Petzold Automated Protein Normalization and Tryptic Digestion on a Biomek-NX Liquid Handler System https://dx.doi.org/10.17504/protocols.io.b3gtqjwn

This is an open access protocol distributed under the terms of the **Creative Commons Attribution License** (https://creativecommons.org/licenses/by/4.0/), which permits unrestricted use, distribution, and reproduction in any medium,

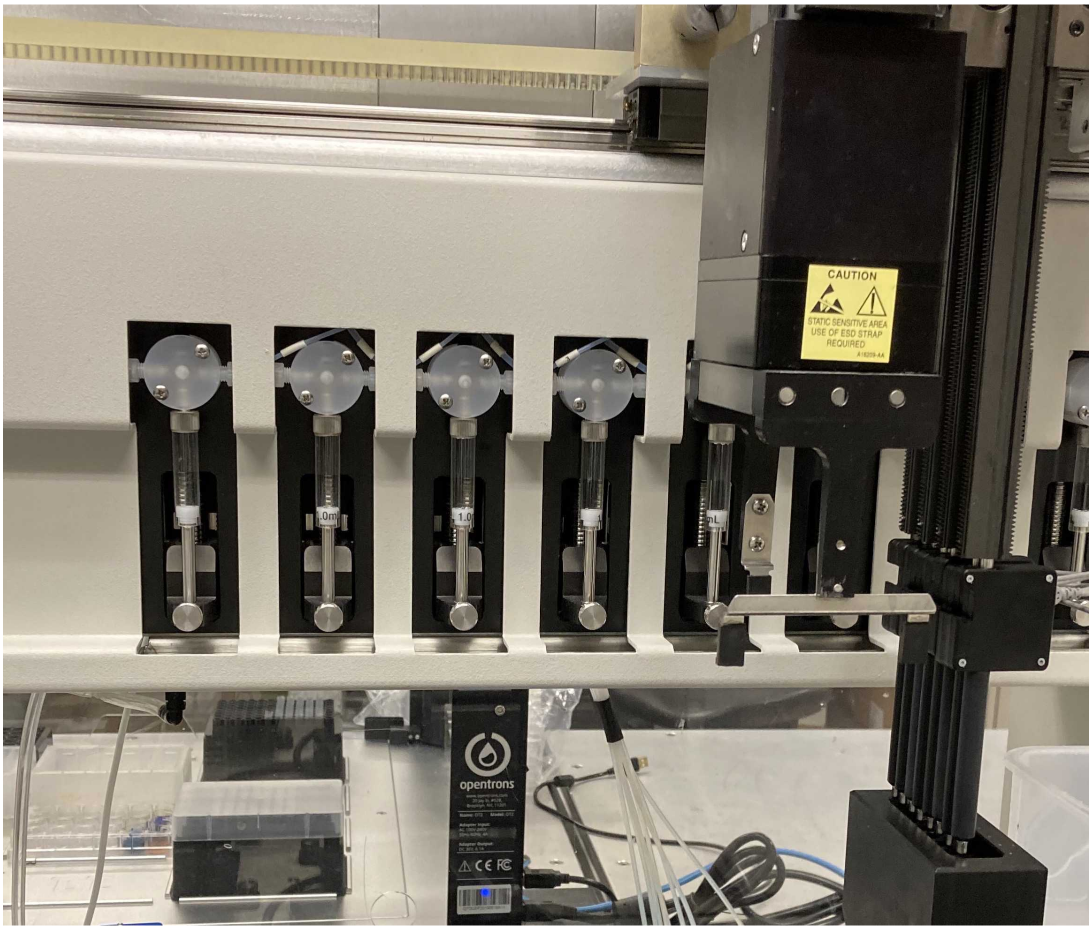


Purging air

## Buffer Transfer

4 Go to "Open Method." Select the "Proteomics" folder and open the method "Proteomics-Normalization Method."

> Because different deck orientations and system components are possible, you will need to modify the method file (attached in the 'Before start' section) for your specific Biomek liquid handler system.

5 Click on "Instrument Setup."



**Citation:** Yan Chen, Tad Ogorzalek, Nurgul Kaplan Lease, Jennifer Gin, Christopher J Petzold Automated Protein Normalization and Tryptic Digestion on a Biomek-NX Liquid Handler System https://dx.doi.org/10.17504/protocols.io.b3gtqjwn

This is an open access protocol distributed under the terms of the **Creative Commons Attribution License** (https://creativecommons.org/licenses/by/4.0/), which permits unrestricted use, distribution, and reproduction in any medium,

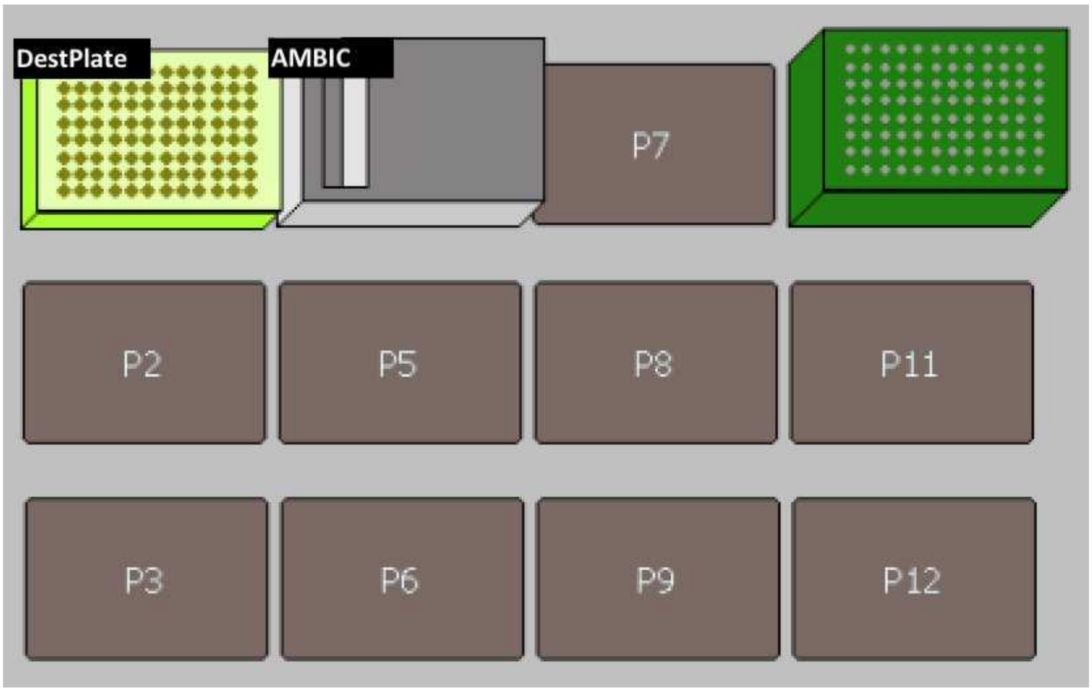


Deck setup

6 Set up the deck (refer to the deck setup picture above):

30s

| A | B | C |
|---|---|---|
| **Deck Label** | **Labware** | **Reagent** |
| **DestPlate** | PCR plate 96-well non-skirted (Thermo Fisher, Cat.#AB0600) on a yellow PCR rack | |
| **AMBIC** | Biomek Reservoir (discontinued) | Ammonium Bicarbonate buffer |
| **tips** | 200 uL pipet tips (Molecular Bioproducts BioRobotix, Cat.#919-262 ) | |

Deck materials

7 Click on "Transfer From File."

8 Copy the **NX-AMBIC.csv** file generated in Excel or via a Jupyter Notebook into the directory that the method is designated to read. For example **C:\Users\jbei\Desktop\Proteomics Methods\CSV files**



**Citation:** Yan Chen, Tad Ogorzalek, Nurgul Kaplan Lease, Jennifer Gin, Christopher J Petzold Automated Protein Normalization and Tryptic Digestion on a Biomek-NX Liquid Handler System https://dx.doi.org/10.17504/protocols.io.b3gtqjwn

This is an open access protocol distributed under the terms of the **Creative Commons Attribution License** (https://creativecommons.org/licenses/by/4.0/), which permits unrestricted use, distribution, and reproduction in any medium,

9 Click on the 2nd "View Datasets" to check that you have copy and pasted the correct volumes in the 96-well format.

10 Click "Finish" to make sure there are no error messages.

11 Click the "Run" button (green arrow) to start. 5m

Protein Transfer

12 Go to "Open Method." Select the "Proteomics" folder and open the method "Proteomics-Plate to Plate Transfer."

13 Click on "Instrument Setup."

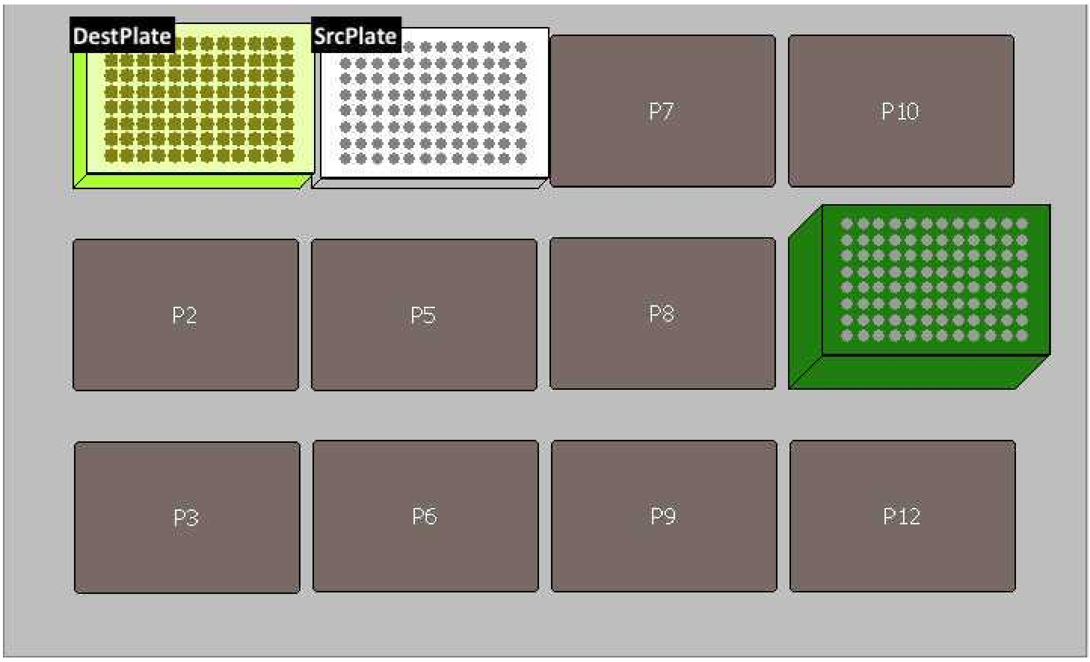


Deck setup

14 Set up the deck (refer to the deck setup picture above): 30s



**Citation:** Yan Chen, Tad Ogorzalek, Nurgul Kaplan Lease, Jennifer Gin, Christopher J Petzold Automated Protein Normalization and Tryptic Digestion on a Biomek-NX Liquid Handler System https://dx.doi.org/10.17504/protocols.io.b3gtqjwn

This is an open access protocol distributed under the terms of the **Creative Commons Attribution License** (https://creativecommons.org/licenses/by/4.0/), which permits unrestricted use, distribution, and reproduction in any medium,

| A | B | C |
|---|---|---|
| **Deck Label** | **Labware** | **Reagent** |
| **DestPlate** | PCR plate 96-well non-skirted (Thermo Fisher, Cat.#AB0600) on a yellow PCR rack | Ammonium bicarbonate buffer |
| **SrcPlate** | PCR plate 96-well non-skirted (Thermo Fisher, Cat.#AB0600) on a yellow PCR rack | protein |
| **tips** | 20 uL pipet tips (Molecular Bioproducts BioRobotix, Cat.#918-262 ) or | |
| | 200 uL pipet tips (Molecular Bioproducts BioRobotix, Cat.#919-262 ) | |

Deck materials

15 Click on "Transfer From File."

16 Copy the **NX-protein.csv** file generated by Excel or via a Jupyter Notebook into the directory that the method is designated to read. For example: **C:\Users\jbei\Desktop\Proteomics Methods\CSV files**

17 Click on "View Datasets" to check that you have copy and pasted the correct volumes in the 96-well format.

18 Click "Finish" to make sure there are no error messages.

19 MANUAL STEP: Use a multichannel pipette to mix protein samples completely right before starting. 5m

20 Click the Run button (green arrow) to start. 8m

## Trypsin Digestion

21 Chemicals to prepare:



**Citation:** Yan Chen, Tad Ogorzalek, Nurgul Kaplan Lease, Jennifer Gin, Christopher J Petzold Automated Protein Normalization and Tryptic Digestion on a Biomek-NX Liquid Handler System https://dx.doi.org/10.17504/protocols.io.b3gtqjwn

This is an open access protocol distributed under the terms of the **Creative Commons Attribution License** (https://creativecommons.org/licenses/by/4.0/), which permits unrestricted use, distribution, and reproduction in any medium,

- Prepare [M]**100 Milimolar (mM) Tris(2-carboxyethyl)phosphine (TCEP) solution** by dissolving **28.7 mg TCEP** in **1 mL 100mM Ammonium Bicarbonate**

- Prepare [M]**200 Milimolar (mM) Iodoacetamide (IAA) solution** by dissolving **36.8 mg Iodoacetamide** in **1 mL 100mM Ammonium Bicarbonate**

- Prepare [M]**1 mg/ml Trypsin** by adding **1 mL 1mM HCl** to **1 mg Trypsin** then vortex to mix

Store TCEP, IAA, and Trypsin in -20C.
IAA is light sensitive. Store in amber tube (Fisher Scientific, Cat.#05-402-31).

22 Add the following reagents to the normalized protein plate, in this order:

1. **2.5 µl 100 mM TCEP**
2. **2.5 µl 200 mM IAA**
3. **1 µl 1 mg/mL Trypsin**

The final concentrations will be 1 µg/µl protein (in 50 µl total volume), 5 milimolar (mM) TCEP, 10 milimolar (mM) IAA , and 1 µl Trypsin (1 mg/ml) (1:50 trypsin:protein ratio). Adjust as needed for your data acquisition protocols.

23 Incubate at **37 °C** for **04:00:00** to **16:00:00** . 20h

## Clarifying Digestion Reaction 15m

24 After digestion, spin at **14000 rpm, 00:15:00** . 15m

25 Pipet supernatant into new PCR plate. Seal and store at **-20 °C** until ready for LC-MS/MS analysis (e.g., [Discovery Proteomics protocol](), [Targeted Proteomics protocol]()).



**Citation:** Yan Chen, Tad Ogorzalek, Nurgul Kaplan Lease, Jennifer Gin, Christopher J Petzold Automated Protein Normalization and Tryptic Digestion on a Biomek-NX Liquid Handler System https://dx.doi.org/10.17504/protocols.io.b3gtqjwn

This is an open access protocol distributed under the terms of the **Creative Commons Attribution License** (https://creativecommons.org/licenses/by/4.0/), which permits unrestricted use, distribution, and reproduction in any medium,

**Citation:** Yan Chen, Tad Ogorzalek, Nurgul Kaplan Lease, Jennifer Gin, Christopher J Petzold Automated Protein Normalization and Tryptic Digestion on a Biomek-NX Liquid Handler System https://dx.doi.org/10.17504/protocols.io.b3gtqjwn

This is an open access protocol distributed under the terms of the **Creative Commons Attribution License** (https://creativecommons.org/licenses/by/4.0/), which permits unrestricted use, distribution, and reproduction in any medium,
